# 1-Cyclo­hexyl-3-{(*E*)-[1-(pyridin-2-yl)ethyl­idene]amino}­thio­urea

**DOI:** 10.1107/S1600536811009317

**Published:** 2011-03-23

**Authors:** Md. Abdus Salam, Md. Abu Affan, Fasihuddin B. Ahmad, Seik Weng Ng, Edward R. T. Tiekink

**Affiliations:** a Faculty of Resource Science and Technology, Universiti Malaysia Sarawak, 94300 Kota Samarahan, Sarawak, Malaysia; b Department of Chemistry, University of Malaya, 50603 Kuala Lumpur, Malaysia

## Abstract

In the title thio­urea derivative, C_14_H_20_N_4_S, the non-ring non-H atoms are approximately planar, with an r.m.s. deviation of 0.0720 Å. The pyridine ring is twisted out of this plane and makes a dihedral angle of 16.85 (13)° with it. The mean plane passing through the cyclo­hexyl ring is almost normal to the central plane [dihedral angle = 69.23 (8)°]. An intra­molecular N—H⋯N(imine) hydrogen bond occurs. Centrosymmetric dimers are formed in the crystal structure *via* pairs of N—H⋯S hydrogen bonds, and these are connected into a supra­molecular chain along the *a* axis *via* C—H⋯π(pyrid­yl) inter­actions.

## Related literature

For related thio­urea structures, see: Tiekink (1989 ▶); Lai & Tiekink (2002 ▶); Muramulla *et al.* (2009 ▶).
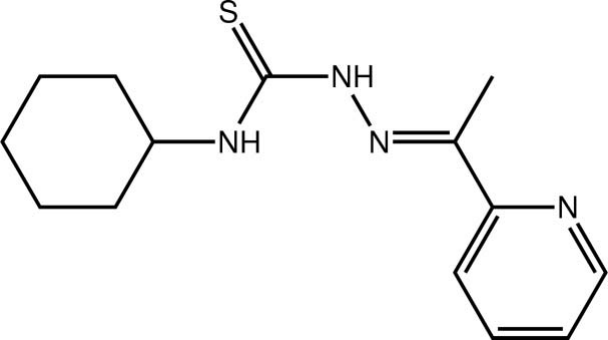


## Experimental

### 

#### Crystal data

C_14_H_20_N_4_S
*M* *_r_* = 276.40
Triclinic, 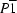

*a* = 5.8824 (6) Å
*b* = 10.2410 (9) Å
*c* = 12.3902 (14) Å
α = 94.718 (8)°
β = 90.427 (9)°
γ = 90.979 (8)°
*V* = 743.74 (13) Å^3^
*Z* = 2
Mo *K*α radiation
μ = 0.21 mm^−1^
*T* = 295 K
0.25 × 0.20 × 0.15 mm

#### Data collection

Agilent Supernova Dual diffractometer with an Atlas detector
Absorption correction: multi-scan (*CrysAlis PRO*; Agilent Technologies, 2010 ▶) *T*_min_ = 0.842, *T*_max_ = 1.000
5817 measured reflections
3292 independent reflections
2355 reflections with *I* > 2σ(*I*)
*R*_int_ = 0.027

#### Refinement

*R*[*F* ^2^ > 2σ(*F* ^2^)] = 0.053
*wR*(*F* ^2^) = 0.151
*S* = 1.04
3292 reflections
181 parameters
2 restraints
H atoms treated by a mixture of independent and constrained refinement
Δρ_max_ = 0.22 e Å^−3^
Δρ_min_ = −0.20 e Å^−3^

### 

Data collection: *CrysAlis PRO* (Agilent Technologies, 2010 ▶); cell refinement: *CrysAlis PRO*; data reduction: *CrysAlis PRO*; program(s) used to solve structure: *SHELXS97* (Sheldrick, 2008 ▶); program(s) used to refine structure: *SHELXL97* (Sheldrick, 2008 ▶); molecular graphics: *ORTEP-3* (Farrugia, 1997 ▶) and *DIAMOND* (Brandenburg, 2006 ▶); software used to prepare material for publication: *publCIF* (Westrip, 2010 ▶).

## Supplementary Material

Crystal structure: contains datablocks global, I. DOI: 10.1107/S1600536811009317/hg5008sup1.cif

Structure factors: contains datablocks I. DOI: 10.1107/S1600536811009317/hg5008Isup2.hkl

Additional supplementary materials:  crystallographic information; 3D view; checkCIF report

## Figures and Tables

**Table 1:** Hydrogen-bond geometry (Å, °) *Cg*1 is the centroid of the pyridyl ring.

*D*—H⋯*A*	*D*—H	H⋯*A*	*D*⋯*A*	*D*—H⋯*A*
N1—H1⋯N3	0.87 (2)	2.16 (2)	2.592 (3)	111 (2)
N2—H2⋯S1^i^	0.88 (2)	2.73 (2)	3.610 (2)	174 (2)
C9—H9*b*⋯*Cg*1^ii^	0.96	2.89	3.776 (3)	155

Symmetry codes: (i) 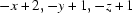; (ii) 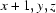.

**Fig. 1.**
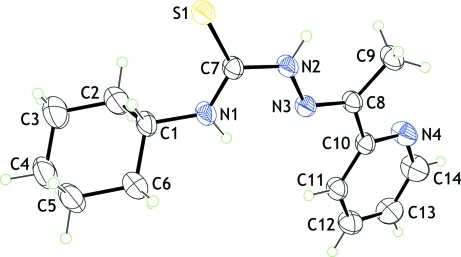
The molecular structure of (I) showing the atom-labelling scheme and displacement ellipsoids at the 35% probability level.

**Fig. 2.**
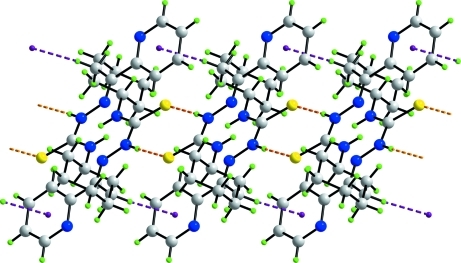
A view of the supramolecular chain aligned along the a axis in (I). The N—H···S hydrogen bonds and C—H···π contacts are shown as orange and purple dashed lines, respectively.

**Fig. 3.**
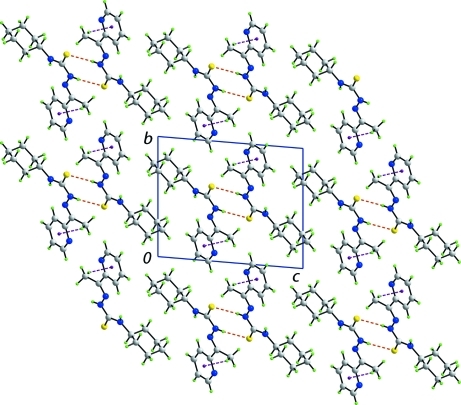
A view in projection down the a axis of the crystal packing in (I). The N—H···S hydrogen bonds and C—H···π contacts are shown as orange and purple dashed lines, respectively.

## supplementary crystallographic information

### Comment

In continuation of long-term structural investigations of thiourea derivatives (Tiekink, 1989; Lai & Tiekink, 2002; Muramulla *et al.*, 2009), the title compound, (I), was investigated. The atoms comprising the thiosemicarbazone backbone of the molecules, *i.e*. S1,N1—N3,C1,C7—C10 are co-planar (r.m.s. = 0.0720 Å). While the pyridine residue is twisted out of this plane as seen in the value of the N3—C8—C10—N4 torsion angle of 164.7 (2) °, the cyclohexyl group is almost normal to the plane; C2—C1—N1—C7 is 87.7 (3) °. The amine-N—H1 and imine-N3 atoms are directed to the same side of the molecule enabling the formation of an intramolecular N—H···N hydrogen bond, Table 1. The pyridine-N atom is directed away from the rest of the molecule and is proximate to the methyl substituent which results in the formation of a C—H···N contact, Table 1.

The crystal packing is dominated by N—H···S hydrogen bonds that lead to centrosymmetric dimers, Table 1. Dimers aligned along the *a* axis are connected into a supramolecular chain *via* C—H···π interactions involving methyl-H and the pyridyl ring. There are no specific intermolecular interactions occurring between chains, Fig. 3.

### Experimental

Cyclohexyl isothiocyanate (0.706 g, 5 mmol) and hydrazine hydrate (0.250 g, 5 mmol), each dissolved in 10 ml ethanol, were mixed with constant stirring. The stirring was continued for 30 min and the white product, *N*(4)-cyclohexylthiosemicarbazide formed was washed with ethanol and dried. A solution of the *N*(4)-cyclohexylthiosemicarbazide (0.51 g, 3 mmol) in 10 ml methanol was refluxed with a methanolic solution of 2-acetylpyridine (0.363 g, 3 mmol) for 5 h after adding 1–2 drops of acetic acid. A white powder separated on cooling the solution which was filtered and washed with methanol. This was recrystallized from methanol and dried *in vacuo* over silica gel. (*M*.pt. 453–455 K; Yield 0.682 g, 76%). Elemental analysis: Calc.: C, 60.83; H, 7.29; N, 11.60%. Found: C, 60.72; H, 7.25; N, 11.57%. FT—IR (KBr, cm^-1^) ν_max_: 3329 (s, NH), 2931, 2851 (s, cyclohexyl), 1580 (w, C═N—N═C), 980 (m, N—N), 1358, 835 (w, C═ S), 657 (m, pyridine in plane).

### Refinement

Carbon-bound H-atoms were placed in calculated positions (C–H = 0.93 to 0.98 Å) and were included in the refinement in the riding model approximation, with *U*_iso_(H) set to 1.2–1.5*U*_eq_(C). The N-bound H-atoms were located in a difference Fourier map and were refined with a distance restraint of N—H 0.88±0.01 Å, and with *U*_iso_(H) = 1.2*U*_eq_(N).

### Crystal data

**Table:**

C_14_H_20_N_4_S	*Z* = 2
*M**_r_* = 276.40	*F*(000) = 296
Triclinic, *P*1	*D*_x_ = 1.234 Mg m^−^^3^
Hall symbol: -P 1	Mo *K*α radiation, λ = 0.71073 Å
*a* = 5.8824 (6) Å	Cell parameters from 2234 reflections
*b* = 10.2410 (9) Å	θ = 2.5–29.3°
*c* = 12.3902 (14) Å	µ = 0.21 mm^−^^1^
α = 94.718 (8)°	*T* = 295 K
β = 90.427 (9)°	Block, colourless
γ = 90.979 (8)°	0.25 × 0.20 × 0.15 mm
*V* = 743.74 (13) Å^3^	

### Data collection

**Table:**

Agilent Supernova Dual diffractometer with an Atlas detector	3292 independent reflections
Radiation source: SuperNova (Mo) X-ray Source	2355 reflections with *I* > 2σ(*I*)
Mirror	*R*_int_ = 0.027
Detector resolution: 10.4041 pixels mm^-1^	θ_max_ = 27.5°, θ_min_ = 2.5°
ω scans	*h* = −7→5
Absorption correction: multi-scan (*CrysAlis PRO*; Agilent Technologies, 2010)	*k* = −12→11
*T*_min_ = 0.842, *T*_max_ = 1.000	*l* = −16→15
5817 measured reflections	

### Refinement

**Table:**

Refinement on *F*^2^	Primary atom site location: structure-invariant direct methods
Least-squares matrix: full	Secondary atom site location: difference Fourier map
*R*[*F*^2^ > 2σ(*F*^2^)] = 0.053	Hydrogen site location: inferred from neighbouring sites
*wR*(*F*^2^) = 0.151	H atoms treated by a mixture of independent and constrained refinement
*S* = 1.04	*w* = 1/[σ^2^(*F*_o_^2^) + (0.0595*P*)^2^ + 0.2389*P*] where *P* = (*F*_o_^2^ + 2*F*_c_^2^)/3
3292 reflections	(Δ/σ)_max_ < 0.001
181 parameters	Δρ_max_ = 0.22 e Å^−^^3^
2 restraints	Δρ_min_ = −0.20 e Å^−^^3^

### Special details

**Table:**

Geometry. All e.s.d.'s (except the e.s.d. in the dihedral angle between two l.s. planes) are estimated using the full covariance matrix. The cell e.s.d.'s are taken into account individually in the estimation of e.s.d.'s in distances, angles and torsion angles; correlations between e.s.d.'s in cell parameters are only used when they are defined by crystal symmetry. An approximate (isotropic) treatment of cell e.s.d.'s is used for estimating e.s.d.'s involving l.s. planes.
Refinement. Refinement of *F*^2^ against ALL reflections. The weighted *R*-factor *wR* and goodness of fit *S* are based on *F*^2^, conventional *R*-factors *R* are based on *F*, with *F* set to zero for negative *F*^2^. The threshold expression of *F*^2^ > σ(*F*^2^) is used only for calculating *R*-factors(gt) *etc*. and is not relevant to the choice of reflections for refinement. *R*-factors based on *F*^2^ are statistically about twice as large as those based on *F*, and *R*- factors based on ALL data will be even larger.

### Fractional atomic coordinates and isotropic or equivalent isotropic displacement parameters (Å^2^)

**Table:**

	*x*	*y*	*z*	*U*_iso_*/*U*_eq_	
S1	0.98373 (10)	0.38035 (6)	0.62998 (5)	0.0566 (2)	
N1	0.6028 (3)	0.4301 (2)	0.73985 (16)	0.0516 (5)	
N2	0.6780 (3)	0.56047 (19)	0.60354 (16)	0.0473 (5)	
N3	0.4954 (3)	0.63316 (18)	0.63973 (15)	0.0443 (4)	
N4	0.2260 (4)	0.9312 (2)	0.61127 (19)	0.0655 (6)	
C1	0.6423 (4)	0.3310 (2)	0.81548 (18)	0.0509 (6)	
H1A	0.7317	0.2612	0.7785	0.061*	
C2	0.7750 (5)	0.3871 (3)	0.9131 (2)	0.0774 (9)	
H2A	0.6945	0.4610	0.9476	0.093*	
H2B	0.9219	0.4189	0.8903	0.093*	
C3	0.8102 (6)	0.2857 (4)	0.9947 (3)	0.1056 (13)	
H3A	0.9038	0.2159	0.9627	0.127*	
H3B	0.8898	0.3264	1.0579	0.127*	
C4	0.5863 (6)	0.2288 (4)	1.0287 (2)	0.0850 (10)	
H4A	0.6135	0.1614	1.0776	0.102*	
H4B	0.4981	0.2969	1.0668	0.102*	
C5	0.4558 (5)	0.1713 (3)	0.9314 (3)	0.0797 (9)	
H5A	0.3094	0.1388	0.9542	0.096*	
H5B	0.5380	0.0976	0.8977	0.096*	
C6	0.4189 (5)	0.2718 (3)	0.8486 (2)	0.0746 (8)	
H6A	0.3421	0.2296	0.7851	0.090*	
H6B	0.3222	0.3407	0.8796	0.090*	
C7	0.7428 (4)	0.4599 (2)	0.66113 (17)	0.0436 (5)	
C8	0.4457 (4)	0.7360 (2)	0.59286 (18)	0.0433 (5)	
C9	0.5690 (4)	0.7871 (2)	0.4999 (2)	0.0575 (6)	
H9A	0.5857	0.7177	0.4437	0.086*	
H9B	0.7165	0.8197	0.5238	0.086*	
H9C	0.4842	0.8567	0.4725	0.086*	
C10	0.2493 (4)	0.8097 (2)	0.64002 (17)	0.0438 (5)	
C11	0.1000 (4)	0.7543 (2)	0.70977 (19)	0.0516 (6)	
H11	0.1192	0.6689	0.7279	0.062*	
C12	−0.0774 (4)	0.8277 (3)	0.7519 (2)	0.0627 (7)	
H12	−0.1794	0.7929	0.7993	0.075*	
C13	−0.1011 (5)	0.9530 (3)	0.7227 (2)	0.0702 (8)	
H13	−0.2191	1.0048	0.7500	0.084*	
C14	0.0518 (5)	1.0001 (3)	0.6527 (3)	0.0768 (9)	
H14	0.0339	1.0850	0.6327	0.092*	
H1	0.478 (3)	0.474 (2)	0.743 (2)	0.065 (8)*	
H2	0.768 (4)	0.579 (3)	0.5503 (14)	0.065 (8)*	

### Atomic displacement parameters (Å^2^)

**Table:**

	*U*^11^	*U*^22^	*U*^33^	*U*^12^	*U*^13^	*U*^23^
S1	0.0544 (4)	0.0507 (4)	0.0685 (4)	0.0198 (3)	0.0197 (3)	0.0228 (3)
N1	0.0532 (11)	0.0492 (12)	0.0560 (11)	0.0178 (9)	0.0174 (9)	0.0209 (9)
N2	0.0480 (10)	0.0408 (11)	0.0557 (11)	0.0121 (8)	0.0150 (9)	0.0165 (9)
N3	0.0448 (9)	0.0368 (10)	0.0527 (11)	0.0086 (7)	0.0091 (8)	0.0101 (8)
N4	0.0693 (13)	0.0387 (12)	0.0918 (16)	0.0159 (10)	0.0248 (12)	0.0197 (11)
C1	0.0587 (13)	0.0454 (13)	0.0517 (13)	0.0179 (10)	0.0168 (11)	0.0172 (11)
C2	0.0801 (19)	0.084 (2)	0.0717 (18)	−0.0111 (16)	0.0005 (15)	0.0302 (16)
C3	0.101 (3)	0.137 (3)	0.087 (2)	−0.018 (2)	−0.0175 (19)	0.062 (2)
C4	0.103 (2)	0.090 (2)	0.0679 (19)	0.0107 (19)	0.0187 (17)	0.0400 (17)
C5	0.088 (2)	0.067 (2)	0.089 (2)	−0.0019 (16)	0.0221 (17)	0.0356 (17)
C6	0.0723 (18)	0.078 (2)	0.0778 (19)	−0.0094 (15)	0.0030 (15)	0.0351 (16)
C7	0.0480 (12)	0.0358 (12)	0.0479 (12)	0.0066 (9)	0.0061 (10)	0.0085 (9)
C8	0.0479 (12)	0.0338 (11)	0.0495 (12)	0.0041 (9)	0.0055 (9)	0.0101 (9)
C9	0.0650 (15)	0.0477 (14)	0.0633 (15)	0.0124 (11)	0.0193 (12)	0.0206 (12)
C10	0.0480 (11)	0.0365 (12)	0.0479 (12)	0.0061 (9)	0.0031 (10)	0.0080 (10)
C11	0.0543 (13)	0.0455 (14)	0.0568 (14)	0.0105 (10)	0.0090 (11)	0.0120 (11)
C12	0.0603 (15)	0.0694 (19)	0.0594 (15)	0.0102 (13)	0.0161 (12)	0.0079 (13)
C13	0.0689 (17)	0.0608 (18)	0.0806 (19)	0.0243 (14)	0.0174 (15)	−0.0027 (15)
C14	0.0823 (19)	0.0418 (15)	0.109 (2)	0.0219 (13)	0.0256 (18)	0.0138 (15)

### Geometric parameters (Å, °)

**Table:**

S1—C7	1.678 (2)	C4—H4A	0.9700
N1—C7	1.332 (3)	C4—H4B	0.9700
N1—C1	1.457 (3)	C5—C6	1.529 (4)
N1—H1	0.870 (10)	C5—H5A	0.9700
N2—C7	1.359 (3)	C5—H5B	0.9700
N2—N3	1.374 (2)	C6—H6A	0.9700
N2—H2	0.878 (10)	C6—H6B	0.9700
N3—C8	1.281 (3)	C8—C10	1.488 (3)
N4—C10	1.331 (3)	C8—C9	1.491 (3)
N4—C14	1.336 (3)	C9—H9A	0.9600
C1—C2	1.503 (4)	C9—H9B	0.9600
C1—C6	1.512 (3)	C9—H9C	0.9600
C1—H1A	0.9800	C10—C11	1.384 (3)
C2—C3	1.523 (4)	C11—C12	1.377 (3)
C2—H2A	0.9700	C11—H11	0.9300
C2—H2B	0.9700	C12—C13	1.370 (4)
C3—C4	1.508 (5)	C12—H12	0.9300
C3—H3A	0.9700	C13—C14	1.364 (4)
C3—H3B	0.9700	C13—H13	0.9300
C4—C5	1.498 (5)	C14—H14	0.9300
			
C7—N1—C1	125.56 (19)	C6—C5—H5B	109.2
C7—N1—H1	114.1 (18)	H5A—C5—H5B	107.9
C1—N1—H1	120.3 (18)	C1—C6—C5	111.2 (2)
C7—N2—N3	118.18 (18)	C1—C6—H6A	109.4
C7—N2—H2	115.9 (18)	C5—C6—H6A	109.4
N3—N2—H2	125.6 (18)	C1—C6—H6B	109.4
C8—N3—N2	119.02 (18)	C5—C6—H6B	109.4
C10—N4—C14	117.7 (2)	H6A—C6—H6B	108.0
N1—C1—C2	111.2 (2)	N1—C7—N2	115.73 (19)
N1—C1—C6	110.3 (2)	N1—C7—S1	124.18 (17)
C2—C1—C6	110.8 (2)	N2—C7—S1	120.08 (16)
N1—C1—H1A	108.1	N3—C8—C10	114.81 (19)
C2—C1—H1A	108.1	N3—C8—C9	126.0 (2)
C6—C1—H1A	108.1	C10—C8—C9	119.19 (19)
C1—C2—C3	111.7 (3)	C8—C9—H9A	109.5
C1—C2—H2A	109.3	C8—C9—H9B	109.5
C3—C2—H2A	109.3	H9A—C9—H9B	109.5
C1—C2—H2B	109.3	C8—C9—H9C	109.5
C3—C2—H2B	109.3	H9A—C9—H9C	109.5
H2A—C2—H2B	107.9	H9B—C9—H9C	109.5
C4—C3—C2	111.2 (3)	N4—C10—C11	122.2 (2)
C4—C3—H3A	109.4	N4—C10—C8	116.23 (19)
C2—C3—H3A	109.4	C11—C10—C8	121.5 (2)
C4—C3—H3B	109.4	C12—C11—C10	119.0 (2)
C2—C3—H3B	109.4	C12—C11—H11	120.5
H3A—C3—H3B	108.0	C10—C11—H11	120.5
C5—C4—C3	110.2 (3)	C13—C12—C11	118.8 (2)
C5—C4—H4A	109.6	C13—C12—H12	120.6
C3—C4—H4A	109.6	C11—C12—H12	120.6
C5—C4—H4B	109.6	C12—C13—C14	118.7 (3)
C3—C4—H4B	109.6	C12—C13—H13	120.7
H4A—C4—H4B	108.1	C14—C13—H13	120.7
C4—C5—C6	111.9 (3)	N4—C14—C13	123.6 (3)
C4—C5—H5A	109.2	N4—C14—H14	118.2
C6—C5—H5A	109.2	C13—C14—H14	118.2
C4—C5—H5B	109.2		
			
C7—N2—N3—C8	173.2 (2)	N2—N3—C8—C10	−178.40 (18)
C7—N1—C1—C2	87.7 (3)	N2—N3—C8—C9	0.0 (3)
C7—N1—C1—C6	−149.0 (2)	C14—N4—C10—C11	0.3 (4)
N1—C1—C2—C3	178.0 (3)	C14—N4—C10—C8	−179.7 (2)
C6—C1—C2—C3	54.9 (3)	N3—C8—C10—N4	164.7 (2)
C1—C2—C3—C4	−56.3 (4)	C9—C8—C10—N4	−13.9 (3)
C2—C3—C4—C5	56.3 (4)	N3—C8—C10—C11	−15.3 (3)
C3—C4—C5—C6	−56.3 (4)	C9—C8—C10—C11	166.1 (2)
N1—C1—C6—C5	−177.8 (2)	N4—C10—C11—C12	−0.7 (4)
C2—C1—C6—C5	−54.2 (3)	C8—C10—C11—C12	179.3 (2)
C4—C5—C6—C1	55.7 (4)	C10—C11—C12—C13	0.4 (4)
C1—N1—C7—N2	−176.6 (2)	C11—C12—C13—C14	0.2 (4)
C1—N1—C7—S1	4.3 (3)	C10—N4—C14—C13	0.3 (5)
N3—N2—C7—N1	8.8 (3)	C12—C13—C14—N4	−0.6 (5)
N3—N2—C7—S1	−172.17 (15)		

### Hydrogen-bond geometry (Å, °)

**Table:**

Cg1 is the centroid of the pyridyl ring [ok as edited?]

**Table:**

*D*—H···*A*	*D*—H	H···*A*	*D*···*A*	*D*—H···*A*
N1—H1···N3	0.87 (2)	2.16 (2)	2.592 (3)	111 (2)
C9—H9C···N4	0.96	2.39	2.822 (3)	107
N2—H2···S1^i^	0.88 (2)	2.73 (2)	3.610 (2)	174 (2)
C9—H9B···Cg1^ii^	0.96	2.89	3.776 (3)	155

Symmetry codes: (i) −*x*+2, −*y*+1, −*z*+1; (ii) *x*+1, *y*, *z*.
